# Viral Population Changes during Murine Norovirus Propagation in RAW 264.7 Cells

**DOI:** 10.3389/fmicb.2017.01091

**Published:** 2017-06-15

**Authors:** Takuya Kitamoto, Reiko Takai-Todaka, Akiko Kato, Kumiko Kanamori, Hirotaka Takagi, Kazuhiro Yoshida, Kazuhiko Katayama, Akira Nakanishi

**Affiliations:** ^1^Laboratory of Radiation Safety, National Center for Geriatrics and GerontologyObu, Japan; ^2^Laboratory of Gastroenteritis Viruses, Virology II, National Institute for Infectious DiseasesMusashimurayama, Japan; ^3^Section of Gene Therapy, Department of Aging Intervention, National Center for Geriatrics and GerontologyObu, Japan; ^4^Division of Biosafety Control and Research, National Institute for Infectious DiseasesTokyo, Japan; ^5^Laboratory of Viral Infection I, Graduate School of Infection Control Sciences, Kitasato Institute for Life Sciences, Kitasato UniversityTokyo, Japan

**Keywords:** norovirus, RAW264.7 cells, cell adaptation, mouse, *Calicivirus*

## Abstract

Laboratory adaptation of viruses is an essential technique for basic virology research, including the generation of attenuated vaccine strains, although the principles of cell adaptation remain largely unknown. Deep sequencing of murine norovirus (MuNoV) S7 during serial passages in RAW264.7 cells showed that the frequencies of viral variants were altered more dynamically than previously reported. Serial passages of the virus following two different multiplicity of infections gave rise to distinct haplotypes, implying that multiple cell-adaptable sequences were present in the founder population. Nucleotide variants lost during passage were assembled into a viral genome representative of that prior to cell adaptation, which was unable to generate viral particles upon infection in cultured cells. In addition, presence of the reconstructed genome interfered with production of infectious particles from viruses that were fully adapted to *in vitro* culture. Although the key nucleotide changes dictating cell adaptation of MuNoV S7 viral infection are yet to be elucidated, our results revealed the elaborate interplay among selected sequences of viral variants better adapted to propagation in cell culture. Such knowledge will be instrumental in understanding the processes necessary for the laboratory adaptation of viruses, especially to those without relevant cell culture systems.

## Introduction

Virus propagation *in vitro* using cultured cells is essential for virology research and for the production of attenuated virus for use in vaccines, including those for measles, polio, and rabies (Minor, 2015); however, the processes shaping viral adaptation to cell culture conditions remain poorly understood.

Norovirus (NoV) is the most prevalent cause of viral gastroenteritis worldwide. The virus belongs to *Caliciviridae* family and consists of a single-stranded, positive-sense, ∼7.6-kb RNA genome that includes a 3′ poly(A) tail. The genome harbors three open reading frames (ORFs)—ORF1 encodes a non-structural (NS) polyprotein that constitutes NS1-2, NS3, NS4, NS5, NS6, and NS7 proteins, whereas ORF2 and ORF3 encode the major and minor capsid proteins VP1 and VP2, respectively. In murine NoV (MuNoV), the accessory viral protein VF1 is encoded by the ORF2 region via an alternative VP1 reading frame (McFadden et al., 2011).

Due to the lack of a permissive cell line, it is currently infeasible to propagate human NoV (HuNoV) using conventional *in vitro* culture systems (Duizer et al., 2004). Limited growth of HuNoV in immunodeficient mice and in human B-cell lines co-cultured with intestinal bacteria has been reported (Taube et al., 2013; Jones et al., 2014). Recent advances in reverse-genetics (Katayama et al., 2014) and development of an enteroid culture system using intestinal stem cells have proven effective for HuNoV propagation *in vitro* (Ettayebi et al., 2016); however, this culture system is impractical for general use owing to its high cost. As such, MuNoV is often used as a surrogate model virus to study NoV because of its ability to proliferate in RAW264.7 cell cultures, as well as murine dendritic cells (Wobus et al., 2004), and of its similarity to HuNoV being infectious agents to gastrointestinal tract. The prototypic MuNoV strain MNV-1 was originally discovered as a lethal agent in *RAG2/STAT1^-/-^* mice (Karst et al., 2003), although MNV-1 infections in wild-type mice caused little pathological changes and were quickly cleared by the host immune system. In comparison, a more prevalent MuNoV stain, CR3, causes persistent infection in the gastrointestinal tract of laboratory mice and is mostly benign to the host, even in immunodeficient mice (Thackray et al., 2007). The MuNoV S7 strain, which is the closest relative to CR3, also appeared to induce no pathological effects (Kitajima et al., 2009); however, whether the strain can cause persistent infection in mice is not known.

Cell adaptation of MNV-1 is associated with attenuated viral pathogenicity in host animals (Wobus et al., 2004). For instance, the V11I and E296K mutations in NS4 and VP1, respectively, are associated with an inability to cause lethal infection in immunodeficient mice, although the potential for growth in RAW264.7 cells appeared unaltered or slightly enhanced (Wobus et al., 2004; Bailey et al., 2008). MNV-1 genomic alterations during cell passages have been well-documented by Sanger sequencing (Wobus et al., 2004; Bailey et al., 2008) and partially by deep sequencing (Mauroy et al., 2016); however, a detailed analysis of these changes in other MuNoV strains and their effect on cell adaptation has yet to be determined.

Here, we report on the detailed examination of MuNoV S7 population changes during cell passage using deep sequencing at two different multiplicity of infections (MOIs). The frequencies of sequence variations were monitored at each passage and linkages analyzed by haplotype reconstruction. Additionally, nucleotide variants lost during cell passage were assembled and used to construct a single genome, which was then examined for its ability to generate infectious particles and possible interactions with genomic sequences fully adapted to *in vitro* propagation. These results revealed dynamic associations between viral population changes in response to cell culture conditions and the complex interplay between viral variants during the selection of sequences better suited for *in vitro* propagation.

## Materials and Methods

### Cells and Viruses

RAW264.7 cells (ATCC TIB-71; American Type Culture Collection, Manassas, VA, United States) were cultured according to the manufacturer’s instructions. The MuNoV S7 strain was kindly provided by Dr. Yukinobu Tohya (Nihon University, Tokyo, Japan) and grown in RAW264.7 cells. Viral preparations derived from cell cultures passaged two or three times were designated as P2 and P3 virus, respectively.

MuNoV infection titers were examined according to the 50% cell culture infective dose (CCID50) as previously described (Katayama et al., 2014). Viral RNA copy number was quantified by real-time reverse transcription polymerase chain reaction (RT-PCR) to approximate viral particle number. Briefly, viral RNA was extracted using QIAamp viral RNA mini kit (Qiagen, Hilden, Germany) and amplified with iScript One-Step RT-PCR kit using SYBR Green (Bio-Rad, Hercules, CA, United States) and the primers, MNV 6082-6108 FW and MNV 6272-6246 RV (Supplemental Table 1), with CFX96 Real-time PCR detection system (Bio-Rad). Fixed amounts of *in vitro*-transcribed MuNoV RNA served as a standard (see “Generation of Recombinant MuNoV”).

The number of viral particles required to achieve one infectious event was assessed using the P13 virus prepared from RAW264.7 cells passaged 10 times after infection with P3 virus. Particle numbers and infectious titers were estimated by quantifying viral RNA copy number and the CCID50, respectively. In our preliminary assessments, ∼500 viral RNA copies were equivalent to one CCID50 unit, although others have shown that the ratio of infectious particles to viral genomes was approximately 1:100 (Baert et al., 2008; Fischer et al., 2015). This five-fold difference could result from differences in quantification method, strain characteristics, or cell culture conditions.

### Sequence Determination of Viral RNA by Sanger Sequencing

Extracted RNA from P2 and P3 MuNoV-containing culture supernatants was resuspended in AVE buffer (Qiagen) and used to generate cDNA in triplicate reactions with either SuperScript III reverse transcriptase (Thermo Fisher Scientific, Waltham, MA, United States) or ReverTra Ace (Toyobo, Osaka, Japan). The resulting cDNA was amplified in triplicate reactions using PrimeStar enzymes (Takara, Otsu, Japan) and five primer sets (Supplemental Table 2), each generating fragments that partially overlapped with the adjacent fragments; thus, nine samples were generated each genomic fragment. The amplified fragments were then separated by gel electrophoresis, isolated, and sequenced with the Big Dye ready reaction kit 3.1 (Thermo Fisher Scientific) and a 3130xl Genetic Analyzer (Thermo Fisher Scientific).

### Preparation of Viral RNA for Next-Generation Sequencing (NGS)

Two different viral populations were generated by passaging the P2 virus in RAW264.7 cells grown in a 6-cm dish. The first was applied to cells at an MOI > 5 and incubated for ∼24 h, at which point many of the cells had died, although more extensive cell death was observed at later passages. Cell supernatant (∼4 mL) was cleared of cell debris by two successive 10 min centrifugations—at 700 ×*g* and then 12,000 ×*g*—at 4°C. The virus was then concentrated by ultracentrifugation with a SW50.1 rotor (Beckman Coulter, Fullerton, CA, United States) at 45,000 rpm for 2 h at 4°C, and the resulting pellet was resuspended in 0.1 mL AVE buffer (Qiagen) for RNA extraction with the QIAamp viral RNA mini kit (Qiagen).

The other viral population was generated by infecting the cells at an MOI of 1 CCID50 (∼500 viral particles)/cell and the supernatant was collected at 48-h post infection (hpi). Cell supernatant (∼4 mL) from each passage was concentrated as described above, the RNA extracted using Isogen II (Takara), and then subsequently resuspended in RNase-free water.

### Reverse Transcription, Double-Stranded DNA Synthesis, Library Preparation, and Deep Sequencing

Viral RNA was used to synthesize cDNA with Superscript III reverse transcriptase (Thermo Fisher Scientific), which was then amplified using PrimeStar GXL enzymes (Takara) and two sets of primers, MNV-1S 14-32/MNV-7A 5361-5380 and MNV-4S 4989-5008/TX30SXN (Supplemental Table 1). The resulting fragments encompassed the proximal and distal halves of the viral genome, respectively, with an overlap of >300 nucleotides. The DNA fragments were gel-isolated and 1 ng used to prepare a cDNA library with the Nextera XT DNA Sample Prep Kit (Illumina, San Diego, CA, United States) according to manufacturer’s instructions. Briefly, DNA was fragmented and tagged by the Nextera XT transposome and then used as a template in a 50-μL, 12-cycle PCR. The amplified DNA was processed as outlined in the Nextera XT protocol and purified with AMPureXP beads (Beckman).

The quality of the purified DNA libraries was assessed on a MultiNA MCE-202 Bioanalyzer (Shimadzu Corporation, Kyoto, Japan). Nucleotide sequencing was performed on an Illumina MiSeq sequencer with a MiSeq Reagent Kit v2 (Illumina) to generate 151 paired-end reads.

### Data Processing and Analysis

Short-reads were trimmed and mapped to the MuNoV reference sequence (GenBank: AB435515.1) using CLC Genomics Workbench 4.65 (CLC Bio, Cambridge, MA, United States) with default alignment settings. BAM files were exported from the CLC Genomics Workbench and analyzed using SAMtools v1.3.1 (Li et al., 2009) to extract sequence coverage and relevant statistics (Supplemental Presentation 1). Major nucleotide variants were called using CLC Genomics Workbench and their frequencies sampled and extracted using SAMtools (Supplemental Table 4). QuasiRecomb version 1.2 (Topfer et al., 2013) was used to reconstruct the viral haplotypes from sequencing data in BAM files. Local haplotype reconstruction across genes was performed using the default setting and conservative parameters to determine the inferred haplotypes and estimate their frequencies.

### Construction of DNA

Unless noted, PrimeStar enzymes (Takara) and the InFusion system (Takara) were used for PCR-fragment generation and fragment insertion into plasmid DNA, respectively. Primers used in this study are listed in Supplemental Table 1. All construct sequences were confirmed by Sanger sequencing.

All genomic variations different from the MuNoV S7 cDNA reference sequence (GenBank: AB435515.1) or PP3 detected by deep sequencing (Supplemental Table 4) were assembled and synthesized as a single artificial genome—termed the PP2 composite sequence (PP2com). The nucleotide differences between PP2com and PP3 spanned from nucleotides 87 to 6752 in the viral genome. The EcoRI and BstBI sites positioned at –73 and 6996 bp in pMNV S7, respectively, (Katayama et al., 2014) were used to replace the original MuNoV sequence with that of PP2com. The 5′-proximal sequence up to the EcoRI site was also included in the synthesis.

pMNV ORF1 PP2, harboring the ORF1 from PP2com, was constructed by exchanging the PP3 sequence between the XhoI and BstBI restriction sites with the ORF2 and ORF3 from pMNV PP2, as well as a small region of the RNA-dependent RNA polymerase-coding sequence that included silent mutations at +4745 and +4877 bp in the genome.

Similarly, pMNV ORF23 PP2, harboring the ORF2 and ORF3 from PP2com, was generated by replacing the pMNV S7 fragment between the XhoI and BstBI restriction sites in with the PP2 sequence from pMNV PP2.

pT7 MNV S7 was generated from pMNV S7 (Katayama et al., 2014) by replacing the EF1-alpha promoter between the SspI and MluI restriction sites and with the T7-promoter sequence. First, two overlapping PCR fragments, A and B, were generated by PCR using pMNV S7 as the template with the primers, SspI FW and T7 MNV RV for A, and MluI RV and T7 MNV FW for B (Supplemental Table 1). The two fragments were then mixed and used as the templates for making insert by PCR using SspI FW and MluI RV as the primers for replacing SspI-MluI fragment of pMNV S7. The resultant pT7 MNVS7 harbors a truncated T7 promoter sequence (5′-TAATACGACTCACTATA-3′) placed proximal to the MNV genomic cDNA sequence for generate RNA with 5′ ends identical to that of natural MuNoV upon *in vitro* transcription.

### Generation of Recombinant MuNoV

Recombinant MuNoV was produced using a plasmid-based reverse-genetics system as described previously (Katayama et al., 2014). The pMNV S7, pMNV PP2, pMNV ORF1 PP2, and pMNV ORF23 PP2 plasmids were transfected individually or in combination into 293T cells cultured in 35-mm dishes by mixing 4 μg DNA, 8 μL P3000 reagent, and 12 μL Lipofectamine 3000 (Thermo Fisher Scientific) in 400 μL Opti-MEM (Thermo Fisher Scientific). Supernatants from transfected cultures were collected 48-h post-transfection and used to infect RAW264.7 cells. The transfected cells were also collected to confirm viral protein expression by Western blotting.

In addition, recombinant MuNoV was also generated using a RNA-based reverse-genetics system as previously described (Arias et al., 2012). Briefly, a series of pT7 MNV constructs was linearized with AscI and used as the template for *in vitro* transcription with the T7 RiboMax express large-scale RNA-production system (Promega, Fitchburg, WI, United States). The synthesized RNA was purified using a Megaclear kit (Thermo Fisher Scientific) followed by 5′-end capping by ScriptCap (Illumina). The capped RNA was then re-purified and used to transfect 293T cells in 35-mm dishes with 4 μg RNA and 8 μL Lipofectamine 2000 (Thermo Fisher Scientific). The viral supernatant was collected 24 h later and used to infect RAW 264.7 cells. The virus was passaged once in RAW264.7 cells to obtain a sufficient titer for cell-infection experiments.

### Western Blotting

Viral proteins from transfected 293T cells were analyzed by Western blotting as previously described (Haga et al., 2016). Briefly, the cell pellet was resuspended in calcium- and magnesium-free Dulbecco’s Phosphate-Buffered Saline (PBS^-^), disrupted by sonication, and centrifuged to remove cell debris. The protein in each sample was quantified and then denatured with SDS-dye buffer (Wako Chemicals, Tokyo, Japan). Aliquots containing about ∼10 μg protein were loaded into each lane, separated by 5–20% SDS-PAGE, and transferred onto a polyvinylidene fluoride (PVDF) membrane using a Trans-Blot system (Bio-Rad). The membrane was blocked with solution containing 2.5% skim milk and 0.5× PVDF blocking buffer (Toyobo), followed by rabbit anti-NS1-2 in solution 1 from the Can Get Signal Kit (Toyobo). After washing, immunoreactive bands were detected with horseradish peroxidase (HRP)-conjugated anti-rabbit IgG antibody in solution 2. Chemiluminescence was detected by ImmunoStar (Wako Chemicals) and recorded using a LAS4000 (GE Healthcare, Little Chalfont, United Kingdom). The membrane was then stripped with Restore Plus Western blot stripping buffer (Thermo Fisher Scientific) and reprobed with HRP-conjugated guinea pig anti-NS7 and mouse anti-actin (Wako Chemicals).

### Immunofluorescent Staining

Supernatants (∼0.5 mL) from cells transfected with MuNoV genome constructs were used to infect RAW264.7 cells grown on coverslips in each well of a 12-well plate. The cells were fixed 48 h later with 4% paraformaldehyde (pH 7.5) and examined for viral protein expression by immunocytochemistry. Mixtures of primary anti-sera, guinea pig anti-VPg, and rabbit anti-VP1 in PBS^-^ supplemented with 0.5% Triton X-100 were used to detect the respective proteins, followed by AlexaFluor 488-conjugated anti-guinea pig IgG and AlexaFluor555-conjugated anti-rabbit IgG secondary antibodies. Samples were embedded in Vectashield mounting medium (Vector Labs., Burlingame, CA, United States) containing 4′,6-diamidino-2-phenylindole (DAPI) and imaged with an epifluorescent microscope (BZ9000; Keyence, Osaka, Japan).

## Results

**FIGURE 1:**
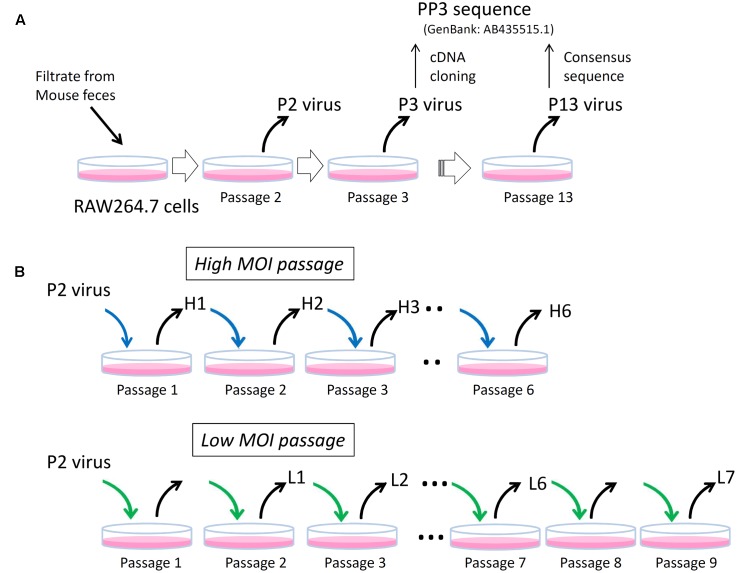
Schematics of experimental layout. **(A)** P2, P3, and P13 viruses. The P2 and P3 viruses are MuNoV preparations passaged in RAW264.7 cells twice (Passage 2) and three times (Passage 3), respectively. Representative molecular clone of MuNoV S7, PP3, was generated by cDNA cloning from P3 virus. After ten passages of P3 virus (Passage 13), which designated as P13 virus, the consensus sequence of the virus became identical to that of PP3. **(B)** High and low MOI passages. Two different MOI conditions were used to inoculate P2 virus and subsequent passages in RAW264.7 cells. The blue and green arrows indicate viral inoculation under MOI > 5CCID50 unit and MOI = 1 CCID50 unit, respectively. Note in “Low MOI passage” number of passages and the samples do not match; no viral samples were taken at passages 1 and 8.

The MuNoV S7 PP3 sequence (GenBank: AB435515.1) originated from a molecular clone obtained from the P3 virus that was passaged three times in RAW264.7 cells (**Figure 1**). The recombinant virus generated from the PP3 sequence by the plasmid-based reverse-genetics system grew well in RAW264.7 cells (Katayama et al., 2014); however, Sanger sequencing revealed that the P3 “consensus” sequence differed by more than 55 nucleotides with 11 amino acid-changes from the PP3 sequence (Supplemental Table 3). Similarly the consensus sequence of P2 virus, which was passaged twice in RAW264.7 cells, differed by 48 nucleotides with 11 missense changes from the reference sequence suggesting that both viral preparations contained pools of heterogeneous sequences (Supplemental Table 3). After 10 passages of the P3 virus in RAW264.7 cells (P13 virus), the consensus viral sequence became identical to that of PP3, thereby confirming that this MuNoV S7 sequence was representative of the one adapted to growth in RAW264.7 cells.

Because earlier passages of MuNoV S7 isolates—including the P2 virus–contained variable clones differing from those of PP3, this suggested that the original viral isolate contained multiple variants, which were further refined by continued propagation in RAW264.7 cells. To further delineate the process by which this occurred, viral sequences from each passage were examined by deep sequencing. For this, viral RNA was prepared from the two set of passages inoculated at different MOIs. The first culture was passaged at an MOI > 5 CCID50/cell, assuming that multiple viral clones would likely coexist in a single cell upon inoculation (high-MOI passage). After six passages, viral RNA was extracted from the infected culture supernatants for sequencing and defined as H1 to H6 for passages one to six. The second culture was inoculated at an MOI of 1 CCID50/cell, assuming that ∼63% of cells were infected and 37% of those were infected with only one virus (a low-MOI passage). It should also be noted that MOIs were only valid at inoculation, as much higher MOI would be expected after single round of viral propagation and release at subsequent infection to adjacent cells. Culture supernatants were harvested over nine passages. Samples L1 to L4 represented those collected from passages two to five, whereas L5, L6, and L7 were from passages six, seven, and nine, respectively.

**FIGURE 2:**
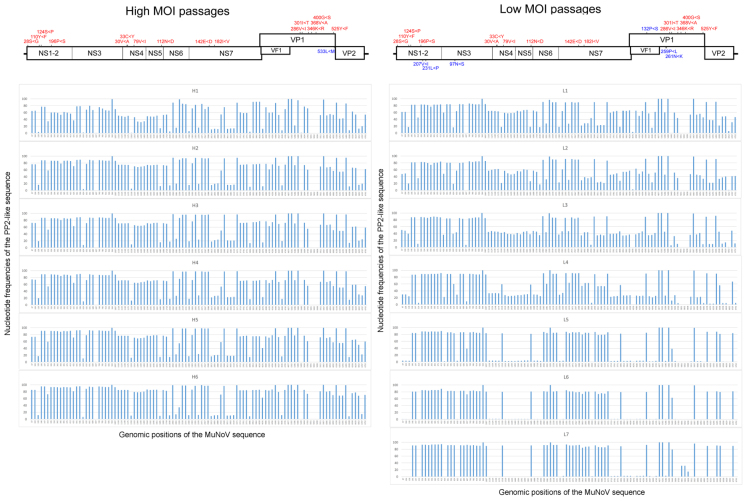
Frequencies of nucleotide differences between the PP2 and PP3 sequences during MuNoV cell passage. Changes in PP2-sequence frequencies during high- and low-MOI passages (**Left** and **Right**, respectively). The horizontal axis indicates the genomic position of the sequences, and the vertical axis indicates the nucleotide frequencies of the PP2-like sequence. Upper panels depict schematics of the MuNoV genome, with indications of non-synonymous nucleotide changes translated to amino acids. Numbers indicate amino acid position affected by the nucleotide variations, and capital letters flanking “<” represent amino acids encoded by PP3 or PP2 sequences, respectively. Letters in red are common changes observed between the high- and low-MOI passages, and those in blue represent changes unique between passages.

Deep sequencing of the viral RNA samples H1–H6 and L1–L7 revealed dynamic sequence changes in the viral population. High- and low-MOI passages showed a total of 89 and 103 nucleotide differences from the PP3 sequence, respectively, of which 87 were shared. The variations included 17 non-synonymous and 22 missense mutations, including 8 shared among the passages (**Figure 2** and Supplemental Table 4). The proportion of non-PP3 sequences—referred to as PP2 sequences—in each passage is summarized in **Figure 2**, with detailed data provided in Supplemental Table 4. Overall changes in nucleotide frequencies in the low-MOI passage were far more evident than those observed with the high-MOI infection, of which many in low-MOI Passage 9 (sample L7) reached nearly zero, indicating that several variants were lost. In contrast, variant frequencies in high-MOI samples showed relatively smaller changes. Moreover, further examination of the low-MOI variants revealed temporal shifts in some nucleotide frequencies (Supplemental Table 4).

To investigate nucleotide frequency linkages, we used QuasiRecomb to generate haplotypes (Topfer et al., 2013). QuasiRecomb implements a hidden Markov model to infer viral quasispecies from deep-coverage NGS data using an expectation-maximization algorithm for maximum posterior-parameter estimation and explicitly accounts for paired-end information. Haplotype reconstructions for the full genome, ORF1, ORF2, and ORF3 are shown in **Table 1A**. Computational processing required deep reads and >1000 coverages were prerequisite for distinguishing single-nucleotide polymorphisms and misreads by deep sequencing (Topfer et al., 2013). Not surprisingly, applying this method to samples H1, L3, and L4 with <1000 coverages on average resulted in large numbers of haplotypes, which likely included misreads. Similar findings were observed with H3 and H4, although it was unknown whether this was attributed to the relatively smaller degree of coverage. Results of comparisons from the remaining samples (H2, H5, and H6 from high-MOI passage and L1 and L4–L7 from low-MOI passage) revealed a general trend of fewer identified haplotypes in low-MOI cultures. Moreover, the ORF1 region generated one or two haplotypes during the final passages of both MOIs, whereas the ORF2 region showed 15 and 2 haplotypes for high- and low-MOI cultures, respectively, which could explain the differences in haplotype number observed for the full genomes (29 and 2 for high- and low-MOI passages, respectively).

**Table 1:** Haplotype frequencies generated by QuasiRecomb.

(A)													
	High-MOI passages						Low-MOI passages						
	H1	H2	H3	H4	H5	H6	L1	L2	L3	L4	L5	L6	L7
**Full genome**													
Average coverage	847	5975	2380	2058	3792	2883	3056	223	227	2295	2506	4032	3148
No. of haplotypes	9966	16	2903	4176	12	29	34	9740	8575	87	1	8	2
Freq. >3%	0	6	0	0	4	5	7	0	0	12	1	2	2
Freq. (1st)	0.0003	0.317	0.009	0.008	0.313	0.54	0.464	0.004	0.001	0.258	1	0.923	0.908
**ORF1**													
Average coverage	871	6018	2464	2098	3829	2950	3266	234	240	2382	2636	4233	3153
No. of haplotypes	8561	8	170	611	10	2	20	5973	5026	54	1	2	1
Freq. >3%	0	7	5	6	6	2	7	0	0	2	1	2	1
Freq. (1st)	0.001	0.438	0.424	0.527	0.446	0.787	0.372	0.002	0.003	0.681	1	0.715	1
**ORF2**													
Average coverage	870	6407	2450	2183	4073	3093	2772	201	201	2118	2303	3728	3217
No. of haplotypes	64	4	215	222	4	15	501	124	8	1	1	1	2
Freq. >3%	6	2	11	7	2	5	0	10	7	1	1	1	2
Freq. (1st)	0.044	0.65	0.06	0.051	0.658	0.691	0.023	0.067	0.379	1	1	1	0.899
**ORF3**													
Average coverage	716	5189	1780	1643	3194	2131	2434	218	215	2284	2269	3662	3243
No. of haplotypes	1	1	1	1	1	1	1	1	2	1	1	1	1
Freq. >3%	1	1	1	1	1	1	1	1	2	1	1	1	1
Freq. (1st)	1	1	1	1	1	1	1	1	0.556	1	1	1	1

**(B)**													
	**High-MOI passages**						**Low-MOI passages**						
	**H1**	**H2**	**H3**	**H4**	**H5**	**H6**	**L1**	**L2**	**L3**	**L4**	**L5**	**L6**	**L7**

**ORF2**													
Average coverage	870	6407	2450	2183	4073	3093	2,772	201	201	2,118	2,303	3,728	3,217
No. of haplotypes	3569	3382	4118	4455	3978	1623	5343	3976	1876	972	131	88	117
Freq. >3%	0	0	0	0	0	1	0	0	0	4	6	7	6
Freq. (1st)	0.004	0.008	0.004	0.004	0.005	0.059	0.002	0.003	0.023	0.101	0.377	0.364	0.334
**ORF3**													
Average coverage	716	5189	1780	1643	3194	2131	2434	218	215	2284	2269	3662	3243
No. of haplotypes	4	4	4	4	4	4	8	4	4	4	4	4	2
Freq. >3%	4	4	4	4	4	3	4	4	2	3	3	3	2
Freq. (1st)	0.486	0.555	0.49	0.421	0.509	0.688	0.369	0.369	0.5	0.67	0.82	0.738	0.924


*QuasiRecomb was used to generate haplotypes of entire genome (Full genome) and individual ORFs, ORF1, ORF2, and ORF3, under conservative setting in **(A)**, and of ORF2 and ORF3 under default setting in **(B)**. Number of average reads of the sequencing data (Average coverage), number of haplotypes (No. of haplotypes), number of haplotypes accounted higher than 3% (Freq. >3%), and frequency of the major haplotype (Freq. 1st) from each passage are shown.*

While the conservative settings only reconstruct major haplotypes and disregard minor ones, the default setting includes minor haplotypes and generates details for changes in haplotype frequencies. However, haplotype reconstructions for ORF1 and the full genome were unsuccessful with the default settings because of inadequate coverage. As such, we only reconstructed the haplotypes with the default setting for ORF2 and ORF3 regions (**Table 1B**). Notably, the ORF2 region harbored a large number of viral haplotypes; 3569 and 5343 in H1 and L1, respectively. In addition, fewer haplotypes were observed in late passages from both MOIs, indicative of haplotype convergence, although more haplotypes were present in H6 as compared to L7. In particular, major haplotype frequencies associated with the ORF2 region observed were 0.691 and 0.059 in H6 and 0.899 and 0.334 for L7 using the conservative and default settings, respectively (**Table 1**). For the ORF3 region, haplotype reconstruction using the conservative settings generated single major haplotypes for the final passages at both MOIs and major haplotype frequencies of 0.688 and 0.924 for the high- and low-MOIs using the default setting (**Table 1**).

**FIGURE 3:**
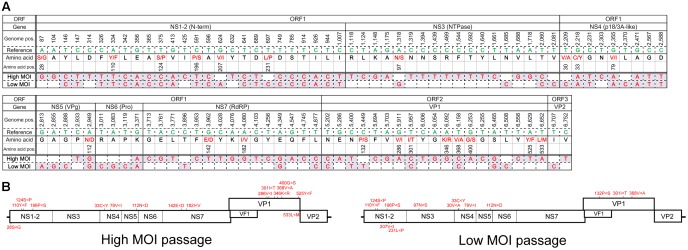
Major haplotype sequences found following the final high- or low-MOI passage. **(A)** Sequence of the major haplotype following the final high- and low-MOI passages. Nucleotides that differed from those found in the PP3 (Reference) sequence are shown along with their genome position (Genome pos.) and the encoded amino acid residue (Amino acid) and their position (Amino acid pos.) in the PP3- or PP2-encoded protein, respectively. Non-synonymous changes are depicted in red. **(B)** Non-synonymous changes of each major haplotype are depicted in the MuNoV genome. Amino acid changes are described similar to those in **Figure 2**.

The major haplotypes identified in the full genome sequences from the final high- and low-MOI passages were distinct—with 81 and 52 nucleotide differences from the original PP3 sequence, respectively, and 35 of which were shared (**Figure 3**). Those changes consisted of 16 non-synonymous and 13 missense mutations, including 8 shared (**Figure 3**) and did not seem localized to a particular region in the viral genome. Notably, the major haplotype sequences from the final passages generated from the full genome and individual ORFs were identical. Moreover, the major haplotypes for ORF2 and ORF3 in the final passages generated by default and conservative settings were identical.

**FIGURE 4:**
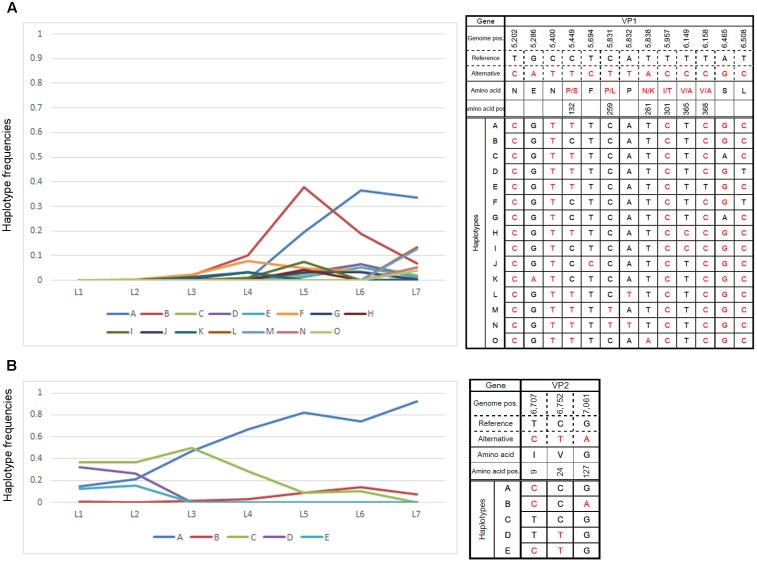
Changes in major haplotype frequencies during each passage. Changes in haplotype frequencies in **(A)** VP1- and **(B)** VP2-encoding regions during low-MOI passages were examined by QuasiRecomb using the default setting. Fifteen haplotypes accounted for >3% of the changes observed in any of the passages were selected and monitored for the frequency of nucleotide changes observed during each passage. The right panels indicate the haplotype sequence along with genome position and possible amino acid change. The left panels show changes in nucleotide frequencies of the selected haplotypes as a line graph. The vertical and horizontal axes indicate the haplotype frequencies and samples for the respective cell passage, respectively.

Close examination of major ORF2 haplotypes in low MOI passage with the default settings revealed dynamic changes in number and frequency. As such, we examined the frequencies of 15 haplotypes that accounted for >3% of those observed in any single passage and found temporal shifts for several haplotypes that were distinct from the majority haplotypes in the final passage (**Figure 4A**). Similar changes in ORF3 haplotype frequencies were also noted (**Figure 4B**).

Genetic variants present in the majority of the viral population in the final passages represented those better adapted for propagation in RAW264.7 cells. Conversely, variants present in earlier passages that were not observed in later passages were likely unfit for growth in RAW264.7 cells. Therefore, viruses harboring PP2-like sequences may represent viral populations not adapted to *in vitro* propagation. We cloned the PP2-like sequences from viral RNA extracted from the PP3 virus and generated recombinant viruses; however, the cloned sequences only covered a minor fraction of the PP2 nucleotide variations, and the recombinant viruses showed no growth defects in RAW264.7 cells (Supplemental Presentation 2).

**FIGURE 5:**
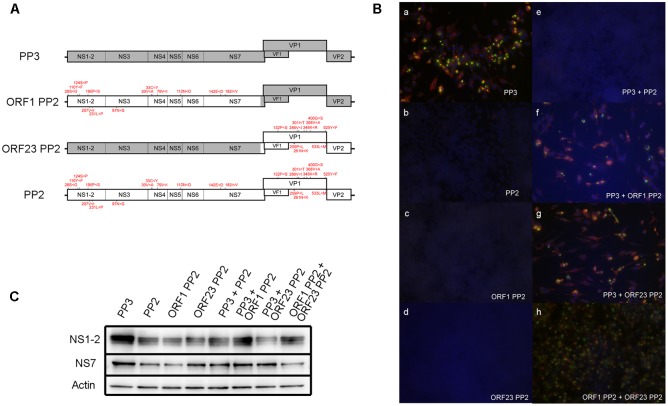
Examination of the ability of the PP2com genome to generate infectious particles. **(A)** Schematic of the recombinant MuNoV genome depicting regions containing PP3 and PP2com sequences. The amino acid residues encoded by the PP2com genome that differed from those encoded by the PP3 genome are indicated in red. **(B)** Supernatant of transfected cells in **(C)** was used to infect RAW264.7 cells. At 48 hpi, cells were fixed and detected for VPg (Green) and VP1 (red) by immunofluorescent detection with staining for DNA by DAPI (Blue) (see “Immunofluorescent Staining” in Materials and Methods). **(C)** Detection of the ORF1 products NS1-2, NS7, and actin by Western blot from the lysates of 293T cells transfected with pMNVs expressing the respective genome. The first four sets of samples were prepared by cotransfecting 2 μg of pT7 MNV S7 and 2 μg each of plasmid expressing the designated recombinant MuNoV genome. The last four sets of the samples were prepared by transfecting the plasmid in combination with those expressing the respective genome.

To determine the cumulative effect of all PP2 nucleotide variations on viral propagation in RAW264.7 cells, we synthesized a PP2 genome that incorporated all nucleotide variations that differed from PP3 detected by deep sequencing (Supplemental Table 4). The PP2com genome was inserted into a PP3 construct previously shown to produce infectious particles upon transfection into 293T cells. Notably, the PP2com viral genome failed to produce infectious particles (**Figure 5B,b**), whereas the PP3 sequence generated high viral titers infectious to RAW264.7 cells (**Figure 5B,a**). Further, conversion of the PP2com ORF1 or ORF23 region to the PP3 sequence was unable to restore particle production (**Figures 5B,c,d**). Subsequent analysis of ORF1 protein expression revealed that NS1-2 and NS7 were detectable in cells transfected with the PP2com sequence (**Figure 5C**); however, VP1 expression was undetectable, given that the extent of sub-genomic replication is low when using the plasmid-based reverse-genetics system (Katayama et al., 2014). Examination of interactions between the PP2com and PP3 sequences showed that the PP2com sequence interfered with the production of infectious particles from the PP3 sequence, as co-transfection of the PP3 and PP2com genomes yielded no infection (**Figure 5B,e**). However, presence of the PP2com ORF1 region alone was insufficient to block the PP3-mediated virus production in one experiment (**Figure 5B,f**), but in two other experiments infectious virus was not produced from PP3 genome with the presence of ORF1 PP2 genome. In contrast, co-transfection of the PP3 genome and ORF23 PP2 generated infectious particles (**Figure 5B,g**), as did that of ORF1 PP3 and ORF23 PP2 (**Figure 5B,h**). The quantification of viral particles in the supernatant of cells transfected with PP3, PP3 + ORF23 PP2, and ORF1 PP2 + ORF23 PP2 contained approximately 2.45 × 10^3^/mL, 2.67 × 10^4^/mL, and 8.09 × 10^3^/mL CCID50, respectively, whereas no particles were found in supernatants from cells transfected with PP3 and ORF1 PP2 cDNA. Collectively, these results indicated that the presence of PP2-like nucleotide variations attenuated viral growth in RAW264.7 cells and interfered with the production of infectious particles from fully adapted sequences. Moreover, variants in the ORF1 region exhibited a *trans-*dominant inhibitory effect; however, co-expression of ORF1 PP2 and ORF23 PP2 to 293T cells generated particles infectious to RAW264.7 cells, indicating that interaction between the PP2com and PP3 sequence was complex—showing both complementation and interference—and dependent upon the context of the sequences.

## Discussion

In this study, we examined details associated with changes in nucleotide frequencies in the MuNoV genome during viral passage following infection with two different MOIs. Interestingly, the number of variants decreased over time depending on the culture conditions, with greater variations generally observed with high-MOI infection. Haplotype analysis revealed that the major haplotype sequences in the final passages differed between high- and low-MOI cultures, suggesting that the initial viral populations contained multiple cell-adaptable sequences. Moreover, the genetic variations lost during in-cell propagation were assembled into a single genome and used to transfect cells, but was unable to produce infectious particles itself, as well as those from cell-adapted PP3. Although the key nucleotide changes dictating cell adaptation in the MuNoV S7 strain have yet to be elucidated and will be examined in future work, our results revealed the elaborate interplay among viral variants to select the sequences better-adapted to propagation in cell culture. To elaborate, our experimental procedures are depicted in **Figure 1**.

Murine norovirus sequences among the known strains are relatively less divergent with mostly fixed genome lengths (Thackray et al., 2007); however, variations in the MuNoV S7 strain exhibit large diversity, indicating the coexistence of multiple haplotypes in the viral isolate. When compared with known nucleotide variations in the MNV-1 (Wobus et al., 2004) and MNV NIH2409 strains (Barron et al., 2011) during cell passage, only two synonymous variations at +1409 and +2081 in MNV-1 were found in the S7 strain (Wobus et al., 2004; Bailey et al., 2008), although no changes associated with enhanced viral growth were detected in this study. Instead, we discovered nearly 100 nucleotide variations—including ∼20 non-synonymous changes, the frequency of which dynamically altered during the viral passages. The nucleotide changes seemed evenly distributed throughout the genome with slight emphasis at the anterior coding region of ORF1 and very few in ORF3 and 3′UTR regions, which contrasted to the results by others reporting high degree of variation at the ORF3 region (Mauroy et al., 2016). Given the limited viability of the PP2com genome (**Figure 5**), these findings indicated that the processes associated with cell adaptation involved multiple rounds of nucleotide alteration or selection.

In this study, we selected viral passages from cells infected at two different MOIs. High-MOI passage might allow for the maintenance of sequence variations and avoid sequence convergence or opportunistic genetic drift that result from genetic bottlenecks because of small subsets in the viral population (Andino and Domingo, 2015). Even under such conditions, haplotype analysis indicated that a single haplotype could flourish to predominate the viral population (**Figure 3**), suggesting the presence of selective pressure for variants better suited for propagation in culture. Not surprisingly, low-MOI passage assumed conditions that resembled a genetic bottleneck wherein the majority of cells do not assume infection with multiple viral clones. Although the secondary infection over the following 48 h period did not warrant conditions limiting entry of infectious clones, sequences exhibited convergence during early phase passages (**Figure 3**) and showed changes in haplotype frequency indicative of genetic drift (**Figure 4**). Genetic drift depends on the diversity in the founder viral population and the number of host cells. Since approximately 1 × 10^6^ cells were used in the present study, the viral populations selected during passage would be no less than 1 × 10^6^. Thus, our observation of viral sequence convergence to a single haplotype likely resulted from selective pressure rather than genetic drift, where sequence selection occurs by chance.

The two different passages generated distinct haplotype sequences that predominated the final viral populations. It is possible that the major haplotypes observed in the low-MOI passage were the product of genetic drift and could differ with prolonged passage. Considering that two distinct haplotypes were observed during the two different passages, and since serial passage of the P3 virus generated a major haplotype identical to PP3, our results suggested the presence of multiple viral haplotypes in the original viral isolate or founder population.

Assuming that PP3 was among the most cell-adapted sequences, we constructed the PP2com genome using sequence variations lost during cell passage. As expected, the PP2com genome was unable to produce infectious particles upon expression using the reverse-genetic system (**Figure 5**). This was not attributed to defects in viral gene expression, as the protein products encoded by ORF1 were expressed at levels comparable with those of PP3. Moreover, the co-transfection of constructs expressing ORF1 PP2 and ORF23 PP2 generated viral particles infectious to RAW264.7 cells (**Figures 5B,C**). Interestingly, co-expression of the PP2com and PP3 genomes blocked the production of infectious particles from PP3, indicating the presence of sequence elements in PP2com that interfered with viral growth *in trans*. Further, presence of the PP2com ORF1 region blocked viral particle production from PP3 genome, but the PP2com ORF23 region only showed a marginal blockade (**Figure 5B,g**). Such effect could be an accumulative since ORF1 and ORF23 from PP2com harbored 81 and 24 nucleotide differences from PP3 sequence, respectively, thus the number of nucleotide differences could be positively correlated with the strength of interference. Such genetic interference could also depend upon the sequence context as co-expression of the ORF1 and ORF23 PP2 genomes generated infectious particles despite that the PP2com-derived ORF1 sequence was present (**Figure 5B,h**). In addition, the coexistence of multiple viral haplotypes has been shown to generate complex interplay, including interference irrespective of coding or non-coding sequences (Ojosnegros et al., 2010; Acevedo et al., 2014). Thus, presence of PP2com-like sequences in the founder viral population would likely impart negative effects on viral propagation *in vitro*.

Here, we used deep sequencing to reveal that the frequencies of MuNoV S7 variants dynamically changed to converge to a cell-adaptable viral sequence during multiple viral passages under cell-culture conditions. The selective process associated with passages of MuNoV S7 in the cultured cells was more complex than previously reported in MNV-1, where only two nucleotide changes were sufficient (Wobus et al., 2004; Bailey et al., 2008). Our findings that the artificial PP2com genome exhibited *trans*-dominant negative effects on viral growth against a cell-adapted genome suggested elaborate interplay among viral variants during the course of the cell-adaptation process in MuNoV. These findings enhanced the understanding of processes related to laboratory adaptation of non-cultivable viruses, especially to those without relevant cell culture systems.

## Author Contributions

KaK and AN designed the study, performed the experiments, and wrote the manuscript. HT, AK, KuK, and RT-T performed the experiments. TK and KY performed bioinformatics analyses. All authors critically revised the manuscript and approved the final version.

## Conflict of Interest Statement

The authors declare that the research was conducted in the absence of any commercial or financial relationships that could be construed as a potential conflict of interest.
