# Supplementary material for: Viral Population Changes during Murine Norovirus Propagation in RAW 264.7 Cells
Source: Front Microbiol. 2017 Jun 15;8:1091. doi: 10.3389/fmicb.2017.01091 (PMC5471328; doi:10.3389/fmicb.2017.01091)

**Supplemental Presentation 1**

**Number of reads in each nucleotide of entire genome sequence in MuNoV S7 by deep sequencing.**

Coverages of deep sequencing are depicted as graph. The horizontal and vertical axes indicate genome position in MuNoV S7 and number of reads in each nucleotide, respectively. The top and bottom panels show coverages of viral sequences in each cell-passage infected with high and low MOI, respectively.

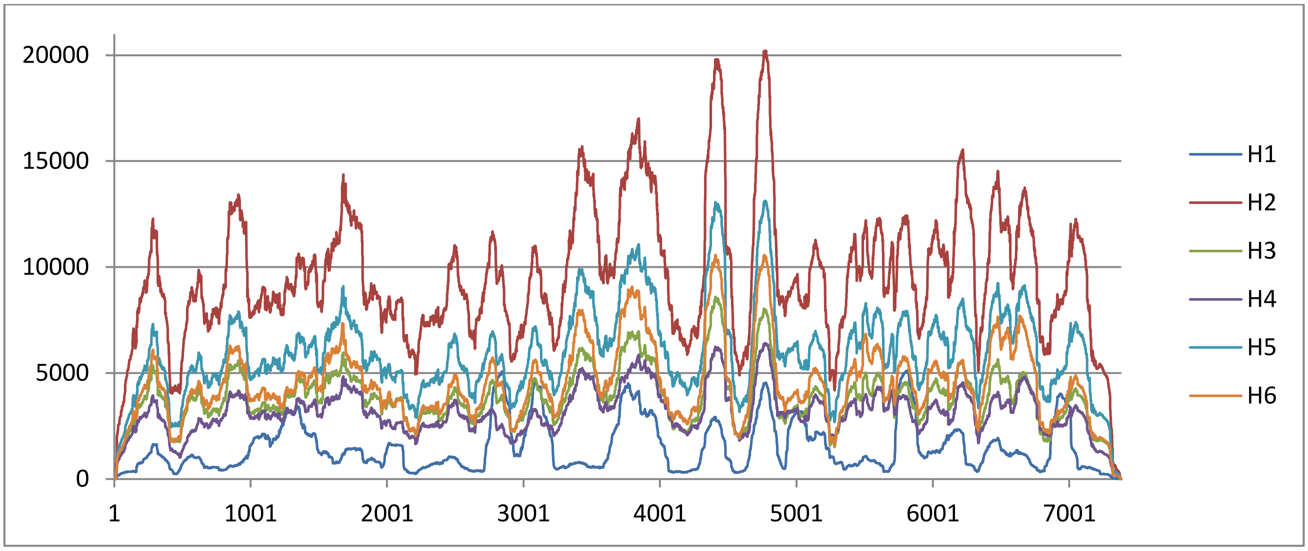


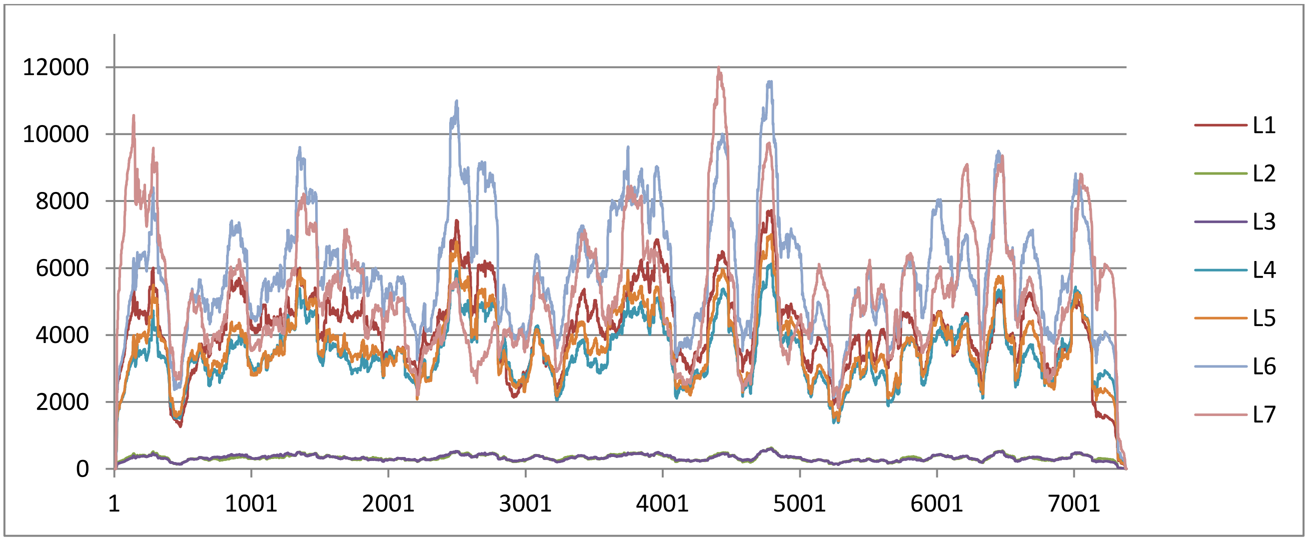

Supplement: Supplementary file 5 [file Presentation_1.pdf]
