# Supplementary material for: Viral Population Changes during Murine Norovirus Propagation in RAW 264.7 Cells
Source: Front Microbiol. 2017 Jun 15;8:1091. doi: 10.3389/fmicb.2017.01091 (PMC5471328; doi:10.3389/fmicb.2017.01091)
Supplement: Supplementary file 6 [file Presentation_2.pdf]

Supplemental Presentation 2

Ability of the recombinant MuNoV bearing cloned PP2 sequences to propagate in RAW264.7 cells.

A. Schematics of the viral variant replaced with the sequence from PP3 to PP2-like sequence cloned from P3 virus (see below, Materials and Methods).

B. Extent of viral RNA in the supernatant (left panel) of and in the RAW264.7 cells (right panel) infected with the recombinant virus.

The vertical axis indicates extent of RNA quantified by real-time RT-PCR. The RNA copy numbers found in the 0.05ml of the supernatant (left panel) or 1/50 of the total RNA extracted from the infected cells (right panel) are shown.

Materials and Methods

Construction of DNA

The MluI-to-XhoI, AflII-BstBI, and XhoI-to-BstBI fragments of pT7 MNV S7 encompassing MNV nucleotide number (n.t.#) 229-4697, 2250-7012, and 4668-7012, respectively, were replaced to the sequence found in the MNV stocks of early passage. The fragments generated by RT-PCR using primers, MluI FW and XhoI RV, AflII FW and BstBI RV, and XhoI FW and BstBI RV (see Table 1), were replaced to that of pT7 MNV S7 using MluI-XhoI, AflII-BstBI, and XhoI-BstBI, restriction sites, generating pT7 MNV MX, pT7 MNV AB, and pT7 MNV XB, respectively.

Generation of recombinant virus, infection to RAW264.7 cells, and quantification of viral RNA.

The recombinant virus bearing the respective sequence was made by RNA-based reverse genetics system as described in the text. The supernatant of RAW264.7 cells infected by the recombinant virus made by transfecting the RNA to 293T cells was extracted for viral RNA by QIAamp viral RNAmini kit (Qiagen) and quantified for viral RNA by real-time RT-PCR as described in text. The viral preparation was adjusted to contain equal concentration of viral RNA and infected to RAW264.7 cells with about 1000 copies per cell. The cells and the conditioned medium was taken at 12, 24 and 36 hours post-infection and extracted for RNA using Isogen II (Takara) and QIAamp viral RNA mini kit (Qiagen), respectively. The viral RNA present in the respective samples was quantified as described in the text.

A

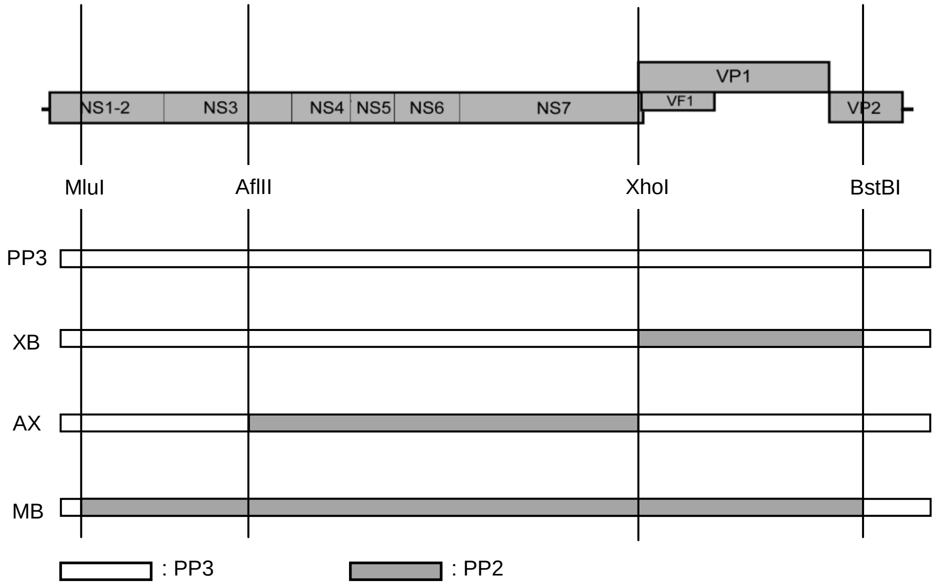
VP1
NS1-2
NS3
NS4
NS5
NS6
NS7
VF1
VP2
MluI
AflII
XhoI
BstBI
PP3
XB
AX
MB
: PP3
: PP2

B

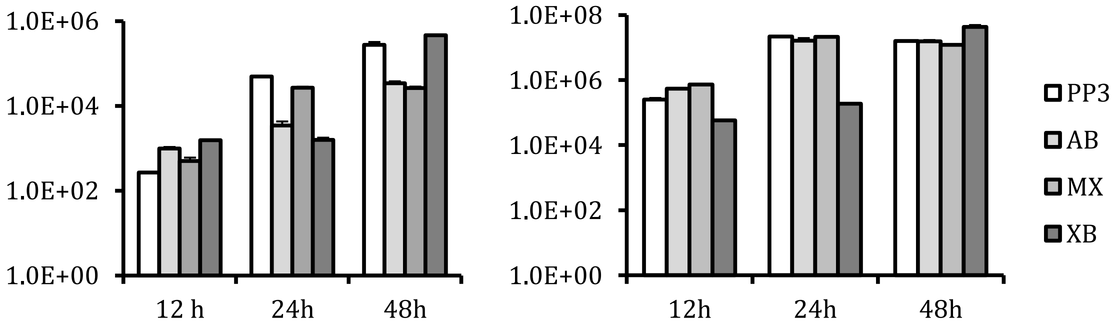
1.0E+06
1.0E+04
1.0E+02
1.0E+00
12 h
24h
48h
1.0E+08
1.0E+06
1.0E+04
1.0E+02
1.0E+00
12h
24h
48h
PP3
AB
MX
XB
